# Validation of Geriatric Care Environment Scale in Portuguese Nurses

**DOI:** 10.1155/2013/426596

**Published:** 2013-05-28

**Authors:** João Paulo de Almeida Tavares, Alcione Leite da Silva, Pedro Sá-Couto, Marie Boltz, Elizabeth Capezuti

**Affiliations:** ^1^Emergency Room, Coimbra University Hospital, Avenida Bissaya Barreto e Praceta Prof. Mota Pinto, 3000-075 Coimbra, Portugal; ^2^Department of Health, University of Aveiro, Campus Universitário de Santiago, 3810-193 Aveiro, Portugal; ^3^Center for Research and Development in Mathematics and Applications, Department of Mathematics, University of Aveiro, Campus Universitário de Santiago, 3810-193 Aveiro, Portugal; ^4^College of Nursing, New York University, 726 Broadway, New York, NY 10003, USA

## Abstract

The number of hospitalized older adults in Portugal necessitates a better understanding of the acute care environment for older adults. This study translated and examined the psychometric qualities of the Geriatric Care Environment Scale (GCES) among 1,068 Portuguese registered nurses (RNs). Four factors emerged from the exploratory factor analyses: resource availability, aging-sensitive care delivery, institutional values regarding older adults and staff, and continuity of care. The internal consistency of the GCES was *α* = .919. The GCES was significantly associated with the variables of region, hospital type, unit type, and RNs perception of hospital educational, staff knowledge, difficulty, rewarding, and burdensome in caring for older adults. Nurses who worked in hospitals centers in the northern region and medical and surgery units had more positive perceptions of the geriatric care environment. More positive perception was also found among RNs that reported more educational support, had more knowledge, and felt more rewarding and less difficulty and burden in caring older adults. This process resulted in a valid and reliable measurement of the geriatric care environment Portuguese version which provides hospital leadership with an instrument to evaluate organizational support for geriatric nursing practice and target specific areas that support or hinder care delivery.

## 1. Introduction

Portugal ranks sixth in the world in terms of its aging population [1]. The 2011 census reported that approximately 19% of the Portuguese population is aged 65 or over [2], whereas they represented 41% of all hospital discharges in 2010 [3]. The average length of stay of older inpatients is 4.5 days, which is longer than inpatients less than 65 years of age (3.7 days) [4]. There is a growing international awareness that the delivery of care to hospitalized older adults is shaped by a combination of factors that includes organizational culture, resource availability, and work environment [5].

Nurses Improving Care for Healthsystem Elders (NICHE) hospitals in North America have recognized the pivotal role of the nurse in improving the hospital outcomes and experiences of older adults [6]. NICHE organizations engage nurses at all levels in multiple roles to transform the geriatric care environment through the use of evidence-based practice guidelines; staff, patient, and family education programs; and evaluation and project management tools [6]. A critical first step in developing system-level initiatives to improve the care of the older adult is the evaluation of the geriatric care environment and the organizational readiness to adopt evidence-based geriatric care. The Geriatric Institutional Assessment Profile (GIAP) instrument is used by NICHE hospitals to evaluate the appropriate use of treatments and the knowledge of geriatric syndromes as well as the organizational attributes of the hospital relevant to geriatric care [6, 7]. The GIAP is a 152-item self-report survey instrument that includes demographic and professional information and eight major scales: perception of geriatric care environment (GCE) [8], 6 subscales about professional issues [9], and a Geriatric Nursing Knowledge/Attitudes Scale [7].

The GCE Scale reflects the growing concern about the work environment and its role in the delivery of care to older people [8, 10–13]. In Portugal, the national Nursing Council has expressed concern about the general nurse work environment without a specific focus on geriatric care [14]. Kim et al. (2009), however, showed a relatively low correlation of the general nursing practice environment (NPE) with the geriatric-specific NPE, underscoring the need to utilize geriatric-specific indices when evaluating the practice environment of nurses caring for older adults [11]. 

Moreover, a “senior-friendly” care environment is needed to attend to the unique needs of older adults during hospitalization [15]. In a senior-friendly hospital the interaction among several factors is recognized: the acute healthcare problem, the developmental phenomena associated with aging, the likelihood of chronic illnesses compounding both diagnosis and treatment, and the physical and social care environment of the hospital [16]. Nurses have an important role to support senior-friendly care environments since they are the largest group of health professionals in the Portuguese hospital settings (87%) [17] and have the most direct and sustained contact with patients. 

A positive GCE is supported by the shared values of leadership and staff that acknowledge the specialized needs of older adults when planning and evaluating treatment. In addition, it fosters the inclusion in decision making of the older persons, family, and nurses (staff training, equipment, supplies, specialized services) and promotes interdisciplinary collaboration [10–12, 18, 19]. Boltz and colleagues (2008) examined the geriatric care environment and its relationship with four of the variables (hospital and unit types, hospital localization, and gender) [18]. They found that the geriatric nursing practice environment has a positive relationship with aging-sensitive care delivery (independent contribution of all three dimensions: resource availability, institutional values, and capacity of collaboration). However, they did not examine the influence of factors concerning RN practice in the care of older adults (perceived hospital educational support and perceived work with older adults as burdensome), which could play an important role in understanding the geriatric care environment. Robinson and Mercer (2007) [20] reported that the perception of lack of knowledge about care older adults among emergency departments' nurses was one of the obstacles to quality of care [20]. The lack of specialized knowledge can be an obstacle to nurses' awareness of the need for positive GCE [21–24]. 

Older adults represent a large percentage of the hospital population in Portugal. Nurses are their main care providers during hospitalization. Consequently, understanding the acute care practice environment (perception of institutional values around care of older adults and staff, interdisciplinary collaboration, and access to geriatric-specific resources) from the perspective of nurses is needed. However, available scales do not exist in Portuguese. Thus, the purpose of this study was to translate and to psychometrically test the Geriatric Care Environment Scale (GCES) in a population of Portuguese registered nurses (RNs) working in diverse hospitals. A second purpose was to evaluate the influence of hospital type and select nurses' perceptions (related to organizational support and burden/rewards) on the GCES and subscales.

## 2. Methods

### 2.1. Data and Sample

The data for this study was collected between February 2011 and May 2011 from RNs at five hospitals that are part of the National Health Care System in the northern and central regions of Portugal. The selection of these hospitals takes in consideration the level of specialization (with acute services such a trauma center or a burns unit, and they have most of medical and surgical specialty units) and hospital structural characteristics (major healthcare facilities of the northern and central regions with a greater number of beds, inpatients, and nurses per hospital), according to the data from the Directorate-General for Health [25]. These characteristics were taken into consideration in order to ensure a diverse sample of responses. The geographical proximity of these hospitals facilitated data collection.

The northern region includes the Greater Oporto metropolitan area and the central region included the cities of Coimbra and Aveiro. Two of the five sites are academic training hospitals for medical and nursing schools (bed number ≥ 1,000), one in the north and the other in the central region. The other three hospitals are considered hospital centers (between 300 and 600 beds). The hospital centers integrate a group of hospitals with a joint administration; this group mainly consists of medical and surgical units. The hospital centers are located in a geographical area covered by an academic hospital that provides the medical, surgical, and critical care units not available in the hospital centers. Two hospital centers are located in the northern region and the other one in the central region. Permission to translate and administer the study instrument was obtained from the NICHE Benchmarking Service of New York University.

This was a nonprobabilistic convenience sample consisting of all registered nurses who worked in medical specialty units (cardiology, internal medicine, nephrology, neurology, oncology, pneumology, and rheumatology), surgical specialty units (general surgery, vascular, maxillofacial and plastic surgery, urology, cardiothoracic surgery, orthopedic surgery, neurosurgery, and burn surgery), and critical care units (intensive care [ICU], coronary, gastroenterology, and the emergency department). We excluded nurses who worked on units serving primarily younger adults or children (e.g., pediatrics, maternity, and hepatic transplants) and nurses' managers and supervisors. 

The researcher (JT), in partnership with the managers of nurses at the units (distributing the surveys), conducted the data collection. The survey was delivered in paper form with envelopes; an online survey was also created for those RNs preferring to answer this way. During the data collection, the researcher was present in all the hospitals and respective units to explain the aim of the study, answer questions, and encourage study participation. Staff placed completed surveys in drop boxes in the units that were collected by the researcher. This project was submitted and approved by the ethics committees of each of the five hospitals: all participants were assured of anonymity and confidentiality. In total, 2,271 surveys were distributed across the five hospitals, and 1,173 were returned, corresponding to an overall response rate of 51.7%, slightly higher than the response rate (40%) reported by McKenzie et al. (2011) in five Canadian hospitals. Of the returned surveys, 105 were excluded (more than two-thirds of the items were missing), and a total of 1,068 surveys were analyzed in this study. 

### 2.2. The Survey

The instrument was the Portuguese version of the GCES of the GIAP, developed by the NICHE program of New York University College of Nursing [7], and entitled “Avaliação do Perfil Geriátrico Institucional” (APGI) in Portuguese. In this study we used the GCES, which has 28 items rated on a 5-point Likert-type scale ranging from 0 to 4 and takes approximately 5 to 10 minutes to complete. Also from the GIAP, five individual investigated-developed questions were included in the analysis. They were concerned with nurses' perception of educational support in their hospital, general knowledge of caring for older adults, and their views on the extent to which they perceived their work with older adults as burdensome. These questions were rated on a 5-point Likert-type scale ranging from 0 to 4. 

Kim and colleagues (2007) [8] conducted an exploratory and confirmatory analysis of the GCES that revealed four factors about nurse perceptions: (1) aging-sensitive delivery (number of items (*k*) = 10,  *α* = 0.94), which relates to the institution's facilities and to aging-sensitive and aging-relevant care for older adults and their families; (2) resource availability (*k* = 8, *α* = 0.90), which relates to access to human and material resources specific to care for older adults and the management support for communication with patients and families; (3) institutional values regarding older adults (*k* = 7, *α* = 0.84), which relates to nurses' perceptions of respect for the rights of older adults, involvement of older adults and families in decision making, and support of nurses' autonomy and personal growth; (4) capacity for collaboration (*k* = 3, *α* = 0.83), which relates to nurses' perceptions of other disciplines' knowledge of geriatric care, use of geriatric protocols, and degree of conflict. The total variance explained by the four factors was 54%. The psychometric results of this scale also yielded good internal consistency [10–12, 18].

For translation and cultural adaptations of the GCES, the principles of the Process for Patient-Reported Outcomes (PRO) Measures Report of the International Society for Pharmacoeconomics and Outcomes Research (ISPOR) Task Force for Translation and Cultural Adaptation were used in this study [26]. The nine steps describing how this was implemented in this study are presented in detail in Table 1. 

### 2.3. Statistical Analysis

In this study, the missing values corresponded to 0.94% of the total sample. An exploratory factor analysis (EFA) was conducted to define the factor structure of the items considered in the GCE Portuguese Scale. The sample size had a subject to item ratio of 38 : 1, thus exceeding the recommendations of others that suggest a subject to item ratio of 10 : 1 or more to perform an EFA [27–30]. As in Kim et al. (2007), the factor solution was obtained using unweighted least squares extraction and Promax rotation [8]. To determine the sampling adequacy, the Kaiser-Meyer-Olkin (KMO) measure and Bartlett's test of sphericity were used. Data were considered adequate for factor analysis when the KMO was greater than 0.5 and the significance of Bartlett's test of sphericity was less than *P* < 0.01 [27–30]. To calculate the number of factors, the latent root criterion (eigenvalues ≥ 1) and scree plot analyses were used. To identify the number of items per factor, we evaluated the factor loading with a cutoff equal to or greater than 0.30, percentage of total variance explained equal to or greater than 40%, and Cronbach's  *α*  equal to or greater than 0.70 [27–30].

For each factor obtained in EFA, a one-way analysis of variance (ANOVA) was conducted to establish statistically significant group mean differences in the GCES scores for selected categorical variables: gender, hospital type, unit type, hospital location (region), perceived hospital educational support, perceived knowledge about caring for older adults, and perceived work with older adults as difficult, rewarding, or burdensome. All these variables are part of the GIAP survey. 

Both normality (by visually inspecting Q-Q plots) and homogeneity (Levene's test) conditions were assessed. Post hoc multiple comparison tests (Turkey's HSD test) were used when ANOVA produced statistically significant results. Spearman's rank test was used to identify possible correlations between the GCES Portuguese subscales and the following continuous variables: age, years of working in their hospital unit, years of working in the institution, and years of experience in the profession. All the statistical analyses were performed using Predictive Analytics Software (PASW) Statistics 18 (IBM Corporation, Armonk, NY), and a *P* value less than 0.05 was considered statistically significant.

## 3. Results 

### 3.1. Description of the Study Sample

The overall majority of the nurses in the sample were female (79.7%), with an average of 34.1 (SD = 8.5). Participants reported an average of 11.3 years (SD = 8.4) of work experience including 10 years (SD = 8.1) working in their institution and 7.5 years (SD = 6.5) working in their units. More than three quarters (83.3%) had a college degree in nursing and one-sixth (16.7%) had a higher level of academic preparation (specialization or a master's/doctoral degree). The majority of the RNs worked at academic hospitals (60.6%), in medical/surgical specialized units (81.2%), as either RNs (88.8%) or specialized nurses (11.2%). Regarding gerontological nursing education, 86.3% of RNs reported not having received any education or training (Table 2).

### 3.2. The Factor Structure of the Portuguese GCES

The significance of Bartlett's test of sphericity (*P* < 0.01) and the value of KMO (0.926) indicated that the sample was adequate to perform EFA. Using the latent root criterion (eigenvalues ≥ 1) a 5-factor solution emerged but the scree plot analysis suggested a 4-factor solution. Analyzing the number of items per factor, the item “it is acceptable to disagree with supervisor regarding care for older adults” was removed because of its low factor loading (<0.30) and because its correlation with the other 27 items was also low. After its removal, a new EFA rotation and extraction were conducted and the results showed a 4-factor solution for both criteria. The 27 items of the Portuguese version of the GCES accounted for 48.09% of the total of variance and had an overall Cronbach's *α*  of 0.919 (Table 3). For the factors, Cronbach's *α*  ranged from 0.894 (for Factor 1) to 0.738 (for Factor 4).

As indicated in Table 3, the first factor, labeled as the “resource availability” subscale, included 11 items with factor loadings ranging from 0.561 to 0.730. The second factor, labeled as “aging-sensitive care delivery” subscale, included 7 items with factor loadings ranging from .583 to 0.804. The third factor, labeled as the “institutional values regarding older adults and staff” subscale, included 6 items with factor loadings ranging from 0.547 to 0.841. The last factor, labeled as “continuity of care” subscale, included 3 items with factor loadings ranging from 0.363 to 0.830. It was labeled as “continuity of care” subscale (see Table 3 for specific statistical information). 

### 3.3. Analysis of the Portuguese GCE Scale and Subscales

In Table 4, we present the results for the total scale and the four subscales. We have categorized the results based on institutional and demographic characteristics as well as on the responses to the five questions. The results for the subscales were significant for all the variables considered (*P* < 0.05).

The association between the subscales was significantly (*P* < 0.05) associated with the variables of hospital type, unit type, and level of agreement with the following: (1) hospital supports education, (2) staff are knowledgeable about older adults care, and (3) older adults care is difficult. Differences in region were statistically significant for the three subscales with the exception of the resource availability subscale (*P* < 0.05). A mixed pattern of responses was found for the variables of gender, older adults care is rewarding, and older adults care is burdensome.

The results for hospital type (see Table 4 for statistical information) reveal that nurses who worked in hospital centers had significantly higher mean scores, indicating that nurses had more positive perceptions of the geriatric care environment. For type of unit, the nurses who worked in critical care units had significantly lower scores than the nurses who worked in the other units, while no statistical differences between those who worked on medical specialty and surgery units were found. Nurses in critical care units had the least positive perceptions of the geriatric care environment. 

Those reporting that they had the least educational support from their hospital also had consistently lower scores than the other groups. A statistically significant difference between the groups was found for the factor “knowledgeable about caring for older adults.” The “very” knowledgeable group had significantly higher values when compared with the other two groups. This is also linked to more positive perceptions about resource availability, aging-sensitive care, institutional values regarding older adults, and the continuity of care for older patients and their families. Nurses who reported more difficulty in caring for older adults also demonstrated significantly lower mean GCES scores when compared to the group with some or no difficulty. 

There were regional differences: compared to nurses in the central region, nurses in the northern region had significantly higher mean values for aging-sensitive care, institutional values regarding older adults, and continuity of care, but not for the resource availability subscale. 

 Nurses who indicated that they felt “very rewarded” in caring for older adults were also more likely to have higher scores in the subscales of aging-sensitive care delivery and institutional values regarding older adults and staff. Similarly, nurses who reported that they felt “very burdened” when caring for older adults had statistically lower mean scores when compared with the other groups for the total GCES score and the subscale of resource availability. Only the resources availability subscale was statistically different for gender: the mean score for males were higher than females, indicating that males had more positive perceptions of the access to human and material resources.

The Spearman's correlation analysis revealed a low correlation between the Portuguese geriatric care environment and the quantitative variables used in the study, ranging from 0.075 to 0.1. 

## 4. Discussion

The Portuguese GCES with four factors is a valid and reliable instrument. The internal consistency values are closer to those found in other studies [8, 10–12, 18, 31], being considered good to very good. However, there are differences when the Portuguese GCES version is compared to the original scale validation. In the Portuguese GCES version the item “it is acceptable to disagree with supervisor regarding care for older adults” was eliminated, and in the original version this item showed a low value (0.33) [8]. Another difference was the reconfiguration of the factors in the Portuguese version with the extraction of the “continuity of care” factor. Also in Portuguese reconfiguration the original subscale “capacity for collaboration” does not appear in our EFA as a factor. The items of this subscale (lack of knowledge, written geriatric policies and procedures, and the difference of opinions among staff) were included in the resource availability subscale. Portuguese RNs understood these items regarding common geriatric problems in our study as a resource for the care of older patients. On the other hand, the factor “continuity of care” was comprised of items previously contained in the aging-sensitive delivery subscale. In contrast to the US where transitional care is a long-standing policy priority [32], the Portuguese healthcare system has only recently emphasized continuity of care between different units, hospitals, and other health and social institutions [4]. The perceptions of Portuguese nurses regarding the care of older adults may have been influenced by these priorities and account for differences in factor structure of the scale. The differences mentioned are most likely attributable to differences in health policy and professional priorities between the countries.

In the Portuguese version, the factor with the greatest percentage variance is the resource availability subscale. This result could be explained by the fact that Portuguese hospitals lack resources, specialized equipment, and services for older adults. Additionally, geriatric care is not yet a priority for organizations, and this aspect is clearly reflected in low scores on institutional values and continuity of care. Given the high proportion of older hospitalized patients, organizations are faced with an imperative to support evidence-based care for older adults and create senior-friendly environments [11, 12, 18]. In general, in this study, nurses were dissatisfied with the geriatric care environment (mean GCES = 1.83 ± 1.09, range of satisfaction with their geriatric care environment = 0–4) compared to the results of Kim and colleagues (2007) (mean GCES = 2.11) [8]. The aspects that emerge as barriers to providing an environment of geriatric care for older people in hospitals are related to resources, the continuity across settings, and the cooperation between managers and nurses in working together. This suggests that the adoption of models of acute care for hospitalized older adults, such as NICHE, in Portugal could promote a change in the work environment to support and improve the geriatric care environment and the delivery of a better quality of geriatric care. As in the United States [18] and Canada [12], there is also considerable room for improvement if an optimal care environment and aging-sensitive care is to be provided in Portugal.

Our findings indicate that the nurses' perceptions of the geriatric care environment (total and subscales) varied by type of hospital in which they worked, thus corroborating the findings of a Canadian study by Mckenzie et al. (2011) [12], but not those of a study by Boltz et al. (2008) [18] which showed that the nurses' perceptions of the geriatric care environment did not vary by type of hospital. However, it is important to note that the studies in the Portuguese and Canadian hospitals examined the perceptions of the geriatric care environment within a universal healthcare system, which is totally different from the United States. Our findings suggest that academic hospitals need to be particularly targeted for initiatives to foster positive geriatric practice environments and the hospital centers could have a higher organizational support and commitment to geriatric care. Further research is needed to understand the influence of universal healthcare on the perception of the geriatric care environment and exactly what kinds of hospital characteristics (centers and academic) impact the nurse practice environment.

In northern Portugal, over the last decade, geriatric content has been introduced to the undergraduate and graduate programs in nursing and other disciplines. The increased knowledge of clinicians in the northern region may contribute to changes in the work environment and RNs' practice and account for their perceptions of the geriatric care environment. However, the resource availability subscale was not associated with the region. This finding is not surprising because budget restrictions have affected Portugal's healthcare in recent years [33].

The nurses' perceptions of the geriatric care environment were more negative in the critical units. A possible explanation is that the nurses in these units are educated to deliver fast-paced critical care to patients in life and death situations and these units could have fewer concerns about GCE that respond to the unique and specific needs relating with older adults health care. Further studies are needed to determine what critical care characteristics influence the geriatric care environment, which should include engagement with the views of nurses, other staff, patients, and families.

The five questions from the GIAP survey are statistically significant for the total GCES and for most of the subscales contributing to the literature that address the geriatric care environment [8, 10–12, 18]. These variables must be addressed in the planning, implementation, and evaluation of initiatives to improve institutional milieu. The findings suggest the importance of hospital managers in supporting the nurses training and education so that they feel equipped to assess and manage challenging issues of older persons care, such as delirium. Furthermore, the nurses would consider their role more rewarding if they understood how to plan, implement, and deliver a better quality of care for older adults and their families. Their perceptions of difficulty in performing their job influenced the nurses' perception of the geriatric care environment. Organizational support should consider the nurses difficulty in caring for hospitalized older adults and identify the characteristics of patients, nurses, and organizational practices that could reduce the difficulty in caring for older adults and promote age-sensitive principles. The significant association between the RNs' perception of the burden and the subscale “resource availability” indicates that nurses who work in hospitals with geriatric-specific resources experience less burden. Our findings underscore the importance of hospitals developing a geriatric nurse education program that could help to improve aging-sensitive care [10, 34, 35] and the nurses' knowledge and care for older adults and positively influence the health outcomes for hospitalized older patients [24, 36, 37]. 

The lack of relationship between the GCES and the variables of age and years of experience (in the institutions and units) is similar to that ones reported by Boltz et al. (2008) [18]. These results indicate that the geriatric care environment can positively influence the delivery of nursing geriatric care with diverse demographic and professional characteristics in a variety of hospitals.

This study has some limitations. Self-completed surveys can lead to bias in the responses of nurses; for example, nurses who are more dissatisfied may be more likely to respond negatively to the GCES. The convenience sample and the hospitals' locations (northern and central regions) may make the generalization of these results difficult, because the size and number of hospitals, their geographic scope, and the number of inpatients are higher in the southern region compared with those for the hospital locations in this study. Additionally, this study has not included nurses from hospitals with fewer than 300 beds, which limits the generalizability of the findings. Our results suggest that although the GCES offers a sound approach to evaluating the care environment for older adults, there is a need to examine the scale structures within diverse geographic and cultural populations. Consequently, rather than comparing the GCES subscale findings between countries, the responses are better viewed within the context of the professional and healthcare priorities environments of each country. Future studies should analyze the geriatric care environment in other country regions and types of hospital. It would also be relevant to understand the relationship of the Portuguese geriatric care environment and geriatric specific patient outcomes (e.g., length of stay, adverse events, and mortality). The association between nurses' perceptions of the geriatric care environment and their experience of the difficulty, burden, and rewarding aspects of caring for older adults should be further investigated. A confirmatory factor analysis to test the invariance of the 4-factor model presented is ongoing.

In conclusion, the translation and cultural adaptations of the GCES confirmed the adequacy of its adaptation for use with Portuguese RNs. We have demonstrated that its psychometric qualities are a reliable measurement of the geriatric care environment, showing good to very good reliability for all the subscales. The validity of the GCES for the Portuguese population allows hospital managers and researchers to have an instrument to evaluate organizational support for geriatric nursing practice. The results of this study can help hospital leadership to target specific areas that support or hinder care delivery. Finally, the findings have revealed the need for undertaking systematic changes in the geriatric care environment in Portugal. This will improve nursing practice and address the complex and specialized needs of the hospitalized older adults. 

## Figures and Tables

**Table 1:** Steps in translation and cultural adaptation.

Step	Activities
(I) Preparation	Permission obtained from the NICHE Benchmarking Service in July 2010. The NICHE research team was invited and involved in this process and helped to clarify any ambiguities and concepts. The adviser of the University of Aveiro supervised the researcher and assisted in the execution of the nine steps. Three experts in geriatric care from Portugal worked closely during the translation process.

(II) Forward translations	Two English translators did independent translations.

(III) Reconciliation	The adviser and one translator analyzed the two translations to define a single forward translation.

(IV) Back translation	An English translator translated the Portuguese version into English.

(V) Back translation review	The questionnaire was reviewed by the adviser and translated to ensure the conceptual equivalence of the translation. The discrepancies were discussed with NYU faculty from the NICHE Benchmarking Service, the adviser and the expert.

(VI) Harmonization	The researchers and the translators shared and defined translation solutions to item discrepancies. The experts reviewed the solutions.

(VII) Cognitive debriefing	The Portuguese translation was tested with 30 RNs from Portuguese hospitals that showed no difficulty in understanding the items in the GIAP.

(VIII) Review of cognitive debriefing results and finalization	The researcher reviewed the results and respondents suggested a few modifications to some words. The researchers and experts agreed with the changes and the translation was finished.

(IX) Proofreading	At the end of the translation, a Portuguese language professor reviewed the Portuguese survey to correct any minor errors in the translation process.

**Table 2:** Nurse demographics and professional characteristics.

Variable	Study sample (*N* = 1068)		
	*N* (%)	M	SD
Age		34.1	8.5
Gender			
Male	217 (20.3)		
Female	851 (79.7)		
Race/ethnicity			
Caucasian	1050 (98.4)		
Other	18 (1.6)		
Marital status			
Single	485 (45.4)		
Divorced	366 (34.3)		
Married	190 (17.8)		
Other	27 (2.6)		
Nursing college degree			
Registered nurses	801 (83.3)		
RN specialist^1^	151 (14.1)		
Master's/doctorate	27 (2.6)		
Professional nursing category			
Registered nurses	949 (88.8)		
Specialized registered nurses	119 (11.2)		
Geriatric education or training			
Never	922 (86.3)		
Short duration courses	94 (8.8)		
Master's/doctorate	52 (4.9)		
Hospital type			
Academic	647 (60.6)		
Hospital centers	421 (39.4)		
Principal unit worked			
Medical units	536 (50.2)		
Surgical units	331 (31.0)		
Critical care	201 (18.8)		
Years of experience in the profession		11.3	8.4
Years of work at the institution		10.0	8.1
Years of work in the current unit		7.5	6.5

^1^The RN specialist is a certificate by the Portuguese's Nursing Council.

**Table 3:** Summary of exploratory factor analysis for the Portuguese Geriatric Care Environment Scale (*N* = 1068).

	F1	F2	F3	F4	Mean ± SD
(1) The older adults omission from care decision*	0.730				1.5 ± 1.0
(2) Economic pressure to limit treatment or length of stay*	0.723				1.4 ± 1.2
(3) Communication difficulties with older adults and their families*	0.719				1.6 ± 1.0
(4) Uncertainty about who is the appropriate decision maker*	0.716				1.6 ± 1.0
(5) Omission of nurses from geriatric care decision*	0.710				1.6 ± 1.2
(6) Staff shortages/time constraints*	0.645				1.0 ± 1.0
(7) Absence of (or insufficient) written geriatric policies and procedures*	0.625				1.7 ± 1.1
(8) Little or no knowledge about care of older adults*	0.622				2.0 ± 1.1
(9) Absence of specialized equipment*	0.603				1.4 ± 1.2
(10) Absence of specialized services for older adults*	0.591				1.4 ± 1.1
(11) Differences of opinion among staff (between disciplines) regarding common geriatric problems*	0.561				2.1 ± 1.0
(12) Staff know how aging affects response to treatment		0.804			2.4 ± 1.1
(13) Staff address geriatric issues		0.799			2.1 ± 1.1
(14) Aging is a factor in care hospitalized older adults		0.791			2.5 ± 1.1
(15) Provide the care that older adults need		0.693			2.1 ± 1.1
(16) Individualized nursing care		0.661			2.4 ± 1.0
(17) Provide the information's that older adults need		0.635			1.8 ± 1.1
(18) Provide the information and support to the families/caregivers		0.583			2.1 ± 1.1
(19) Engagement of staff in the geriatric care			0.841		2.0 ± 1.1
(20) The staff protects the rights of older adults			0.703		2.3 ± 1.1
(21) Personal growth is stimulated			0.630		1.9 ± 1.2
(22) Respect older adults in caring older adults			0.615		2.6 ± 1.2
(23) The geriatric policies and guidelines are established based on the inputs of the staff			0.610		1.5 ± 1.1
(24) Clinicians and administrators work together to solve older adults' problems			0.547		1.3 ± 1.0
(25) Continuity of care between hospitals is adequate				0.830	1.5 ± 1.1
(26) Continuity of care between settings is adequate				0.562	2.0 ± 1.8
(27) Baseline information is obtained at hospital admission				0.363	1.6 ± 1.8

Eigenvalue	8.81	3.25	1.69	1.19	
% variance	30.79	10.12	4.41	2.77	48.09
Cronbach's *α*	0.894	0.885	0.827	0.738	0.919

F1: resource availability; F2: aging-sensitive care; F3: institutional values regarding older adults and staff; F4: continuity of care. *Reverse-scored item.

**Table 4:** Portuguese Geriatric Care Environment Scale (GCES) subscales and total scores (*N* = 1068).

Variable (*N*)	F1		F2		F3		F4		Total score	
	M ± SD	*F* (*P*)	M ± SD	*F* (*P*)	M ± SD	*F* (*P*)	M ± SD	*F* (*P*)	M ± SD	*F* (*P*)
Gender										
Male (217) Female (851)	18.2 ± 8.4 16.9 ± 8.3	4.4 (0.036)	15.3 ± 5.8 15.8 ± 5.9	1.2 (0.269)	11.3 ± 5.2 11.6 ± 4.9	0.5 (0.476)	5.2 ± 2.7 5.1 ± 2.7	0.3 (0.585)	50.5 ± 17.1 48.8 ± 16.7	1.8 (0.184)
Hospital type										
Academic (647) Centers (421)	16.3 ± 8.2 18.5 ± 8.4	17.4 (<0.001)	14.8 ± 6.2 16.4 ± 5.2	20.7 (<0.001)	11.0 ± 5.0 12.3 ± 4.8	16.7 (<0.001)	4.8 ± 2.7 5.6 ± 2.5	21.5 (<0.001)	46.9 ± 17.0 51.7 ± 15.8	31.1 (<0.001)
Unit type										
Critical care (201) Medical (536) Surgery (331)	15.3 ± 9.0 17.4 ± 8.2 17.9 ± 8.1	6.7 (0.001**)	12.0 ± 6.8 16.1 ± 5.3 16.3 ± 5.4	46.6 (<0.001**)	9.2 ± 4.8 12.0 ± 4.8 12.1 ± 4.9	29.4 (<0.001**)	3.9 ± 2.7 5.4 ± 2.6 5.5 ± 2.4	29.9 (<0.001**)	40.1 ± 19.2 50.9 ± 15.6 51.8 ± 15.2	38.4 (<0.001**)
Region										
North (375) Center (693)	17.5 ± 8.2 17.0 ± 8.4	0.7 (0.388)	16.5 ± 5.5 14.8 ± 6.0	21.1 (<0.001)	12.9 ± 4.9 10.7 ± 4.8	49.9 (<0.001)	5.5 ± 2.6 4.9 ± 2.7	14.2 (<0.001)	52.4 ± 15.9 47.4 ± 17.0	21.3 (<0.001)
Perception										
Hospital educational support										
Less (729) Adequate (276) More (63)	15.9 ± 8.1 19.5 ± 8.1 21.9 ± 8.5	30.9 (<0.001**)	14.3 ± 5.8 17.6 ± 5.2 19.5 ± 4.0	52.7 (<0.001*)	10.4 ± 4.7 13.6 ± 4.6 15.6 ± 4.1	72.7 (<0.001*)	4.7 ± 2.6 6.0 ± 2.6 7.1 ± 2.1	46.3 (<0.001*)	45.1 ± 16.1 56.5 ± 14.9 66.1 ± 13.4	82.7 (<0.001*)
Staff knowledge of older adults care										
Not very (82) Somewhat (523) Very (463)	14.8 ± 7.8 16.1 ± 8.2 18.6 ± 8.4	13.1 (<0.001***)	13.0 ± 5.9 14.8 ± 5.9 16.5 ± 5.6	17.8 (<0.001*)	9.5 ± 4.9 10.9 ± 4.9 12.6 ± 4.8	21.9 (<0.001**)	4.7 ± 2.8 4.8 ± 2.7 5.5 ± 2.5	10.4 (<0.001***)	43.1 ± 17.0 46.5 ± 16.6 53.2 ± 16.1	26.2 (<0.001***)
Difficulty of caring for older adults										
Great (662) Some or not (406)	15.9 ± 8.0 19.2 ± 8.6	41.3 (<0.001)	14.6 ± 5.9 16.7 ± 5.5	33.7 (<0.001)	10.9 ± 5.0 12.4 ± 4.7	23.0 (<0.001)	4.9 ± 2.6 5.5 ± 2.6	15.708 (<0.001)	46.3 ± 16.7 53.9 ± 15.8	53.0 (<0.001)
Reward of caring for older adults										
Not (744) Very (324)	16.90 ± 8.0 17.8 ± 9.0	2.4 (0.121)	15.0 ± 5.7 16.3 ± 6.1	11.5 (0.001)	11.1 ± 4.7 12.4 ± 5.5	14.1 (<0.001)	5.0 ± 2.6 5.4 ± 2.8	3.7 (0.053)	48.0 ± 16.2 51.8 ± 18.0	11.1 (0.001)
Of burden of caring older adults										
Very (455) Somewhat (545) Not (68)	15.6 ± 7.8 18.2 ± 8.5 18.7 ± 9.2	13.8 (<0.001**)	15.1 ± 5.6 15.6 ± 5.9 15.4 ± 7.0	0.9 (0.386)	11.4 ± 4.9 11.5 ± 4.9 11.6 ± 5.4	0.3 (0.774)	4.9 ± 2.6 5.3 ± 2.7 5.5 ± 2.7	2.6 (0.070)	47.7 ± 15.7 50.7 ± 17.3 51.3 ± 19.0	6.3 (0.002**)

F1: resource availability; F2: aging-sensitive care; F3: institutional values regarding older adults and staff; F4: continuity of care.

*Each group differed from the other two (Turkey's HDS test, *P* < 0.05). **The first group differed from the second and third groups (Turkey's HDS test, *P* < 0.05). ***The third group differed from the first and second groups (Turkey's HDS test, *P* < 0.05).
